# Holevo Capacity of Discrete Weyl Channels

**DOI:** 10.1038/s41598-018-35777-7

**Published:** 2018-11-29

**Authors:** Junaid ur Rehman, Youngmin Jeong, Jeong San Kim, Hyundong Shin

**Affiliations:** 1 Department of Electronic Engineering, Kyung Hee University, 1732 Deogyeong-daero, Giheung-gu, Yongin-si, Gyeonggi-do 17104 Korea; 2 Department of Applied Mathematics and Institute of Natural Sciences, Kyung Hee University, 1732 Deogyeong-daero, Giheung-gu, Yongin-si, Gyeonggi-do 17104 Korea

## Abstract

Holevo capacity is the maximum rate at which a quantum channel can reliably transmit classical information without entanglement. However, calculating the Holevo capacity of arbitrary quantum channels is a nontrivial and computationally expensive task since it requires the numerical optimization over all possible input quantum states. In this paper, we consider discrete Weyl channels (DWCs) and exploit their symmetry properties to model DWC as a classical symmetric channel. We characterize lower and upper bounds on the Holevo capacity of DWCs using simple computational formulae. Then, we provide a sufficient and necessary condition where the upper and lower bounds coincide. The framework in this paper enables us to characterize the exact Holevo capacity for most of the known special cases of DWCs.

## Introduction

One of the fundamental tasks in the context of information theory is to compute the maximum rate at which information can be reliably transmitted^1,2^. Classical channels have the capability of transmitting classical information only. On the contrary, quantum channels are more rich in terms of communication tasks^3,4^. Trivially, quantum channels are capable of transmitting quantum information. However, due to the versatile nature and unique features of quantum mechanics, it is possible to associate multiple communication tasks with a quantum channel^5^. Thus, we have classical capacity, quantum capacity, private classical capacity, and entanglement-assisted classical capacity of a quantum channel. All of theses correspond to different information communication tasks^6–9^.

The calculation of various capacities involves an optimization task that is not easy to perform. For example, the capacity of a classical channel is given by a single letter formula—the mutual information between input and output of the channel—maximized over the probability distribution of the input random variable^10^. Efficient methods exist that can perform this maximization^11,12^. On the contrary, capacities (except the entanglement-assisted classical capacity) of a quantum channel are given in terms of regularization of asymptotically many channel uses. These regularized formulae are mathematically intractable in general and put forth an unsolvable optimization problem^13^. Simplification of these formulae is not possible due to the nonadditive and nonconvex natures of capacities of quantum channels^14–16^. The need of regularization, however, can be removed either (1) if the capacity of the channel is additive, or (2) if we restrict the optimization to be on the individual channel use. For example, unital qubit channels^17^ and entanglement breaking channels^18^ are known to be additive and thus their classical capacity can be computed without the need of regularization. Similarly, for the task of classical communication over a quantum channel, one can prohibit the use of inputs states correlated over multiple uses of the channel–effectively allowing optimization on the individual channel use only–to obtain a lower bound on the classical capacity of a quantum channel. This notion of capacity is known as the Holevo capacity. Even with such a simplification of the problem, the calculation remains considerably demanding. As a matter of fact, calculation of the Holevo capacity falls in the category of NP-complete problems^15,19^.

This multilayer difficulty has stimulated a good amount of research in the field of quantum information theory. Different researchers have taken different routes to accomplish this seemingly impossible task. For example, different definitions of capacities have been proposed^20^, analytical expressions for the special channels have been found^21^, and some bounds that are additive and easier to calculate have been computed^22^ to solve the problem of regularization. While for solving the difficulty of calculation, exploiting special properties of a given channel^23^, and methods that can approximate the capacity upto a fixed a posteriori error have been proposed^24^.

In this work we give easy to compute lower and upper bounds on the Holevo capacity of discrete Weyl channels (DWCs). Our employed approach involves modeling the DWC as a classical symmetric channel and using the existing results from the classical information theory to lower bound the Holevo capacity of a DWC. The upper bound is based on the majorization relation of any possible output state of a DWC with the most ordered state based on the channel parameters. We give a necessary and sufficient condition for which the two bounds coincide. We find that this condition is met for the known special cases (Pauli qubit channel, and the qudit depolarizing channel) of DWC and hence we can recover the exact capacity expression for these cases. Through numerical examples we show that the coincidence of two bounds is sufficient but not necessary for the lower bound to give exact capacity.

## Discrete Weyl Channel

A quantum state ***ρ*** on the Hilbert space is a positive operator with unit trace (i.e., a density operator). We consider the Hilbert space of finite dimension *d*. The state is said to be *pure* if it has the form ***ρ*** = |*ψ*〉 〈*ψ*|. We usually denote a pure state simply by a ket e.g., |*ψ*〉, which is a column vector in the Hilbert space. A quantum channel ${\mathscr{N}}:{\boldsymbol{\rho }}\to {\mathscr{N}}({\boldsymbol{\rho }})$ is a completely positive trace preserving (CPTP) map transforming the input state ***ρ*** to an output state ${\mathscr{N}}({\boldsymbol{\rho }})$. The map can be specified in terms of Kraus operators {***A***_*i*_} as ${\mathscr{N}}({\boldsymbol{\rho }})={\sum }_{i}\,{{\boldsymbol{A}}}_{i}{\boldsymbol{\rho }}{{\boldsymbol{A}}}_{i}^{\dagger }$ where ${\sum }_{i}\,{{\boldsymbol{A}}}_{i}^{\dagger }{{\boldsymbol{A}}}_{i}={{\boldsymbol{I}}}_{d}$ and ***I***_*d*_ is the identity operator on the *d*-dimensional Hilbert space. For a random unitary channel, it is possible to represent Kraus operators as ${{\boldsymbol{A}}}_{i}=\sqrt{{p}_{i}}{{\boldsymbol{B}}}_{i}$, such that the channel applies an operator ***B***_*i*_ on the input state with the probability *p*_*i*_^25^.

Let ${{\boldsymbol{\sigma }}}_{0}={{\boldsymbol{I}}}_{2}$ be the 2 × 2 identity matrix, and

1 $${{\boldsymbol{\sigma }}}_{1}=[\begin{array}{cc}0 & 1\\ 1 & 0\end{array}],\,{{\boldsymbol{\sigma }}}_{2}=[\begin{array}{cc}0 & -i\\ i & 0\end{array}],\,{{\boldsymbol{\sigma }}}_{3}=[\begin{array}{cc}1 & 0\\ 0 & -1\end{array}]$$

be the Pauli matrices. The Pauli qubit channel, denoted by ${{\mathscr{N}}}_{{\rm{p}}}({\boldsymbol{\rho }})$, is then defined as

2 $${{\mathscr{N}}}_{{\rm{p}}}({\boldsymbol{\rho }})=\sum _{i\mathrm{=0}}^{3}\,{p}_{i}{{\boldsymbol{\sigma }}}_{i}{\boldsymbol{\rho }}{{\boldsymbol{\sigma }}}_{i}^{\dagger }$$

which is a random unitary channel.

**Figure 1:**
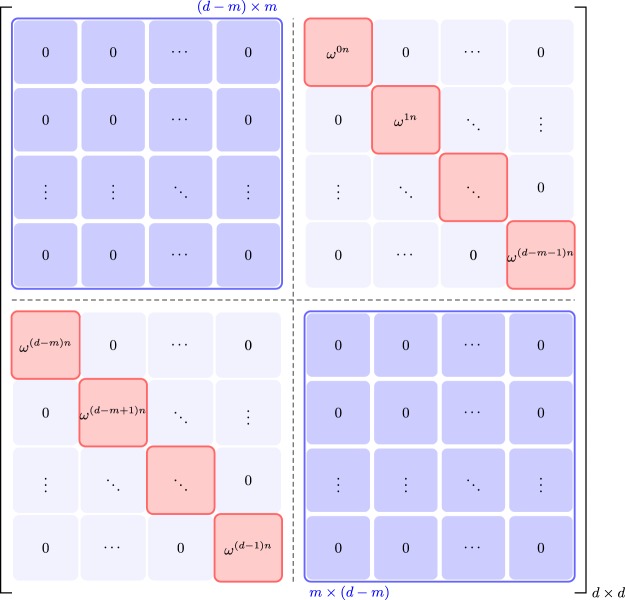
The general structure of a Weyl operator ***W***_*nm*_ in an arbitrary dimension *d*.

Discrete Weyl operators are a non-Hermitian generalization of Pauli operators for dimension *d*^26^. A Weyl operator ***W***_nm_ on the *d*-dimensional Hilbert space is defined as^27^

3 $${{\boldsymbol{W}}}_{nm}=\sum _{k\mathrm{=0}}^{d-1}\,{\omega }^{kn}|k\rangle \,|(k+m)\,{\rm{mod}}\,d\rangle $$

for $n,\,m=\mathrm{0,}\,\mathrm{1,}\,\cdots ,\,d-1$; $\omega =\exp (2\pi \iota /d)$; and |*k*〉 is the *k*th basis vector in the computational basis (for notational convenience, the indexing of entries of vectors and matrices start from 0). A general structure of a *d*-dimensional Weyl operator ***W***_*nm*_ is shown in Fig. 1.

### Property 1.

*A Weyl operator* ***W***_*nm*_*, when applied on a d-dimensional vector α, up-shifts the entries of α by m locations and rotates ith entry (according to new indexing) by a phase of ω*^*in*^*. We refer to this property as shift and phase operation of Weyl operators*.

**Figure 2:**
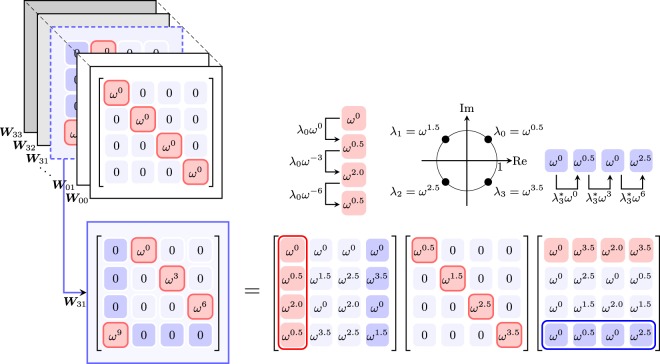
A schematic illustration for the structure of discrete Weyl operator ***W***_31_ on a 4-dimensional Hilbert space. Each eigenvalue *λ*_s_ and eigenvector |*λ*_s_〉 can be found using (4) and (30), respectively.

Eigenvalues of a Weyl operator ***W***_*nm*_ are given by (see supplementary material),

4 $${\lambda }_{s}={\omega }^{mn\frac{(d-1)}{2}+s}$$

where $s\in \{(mk-nj)\,{\rm{mod}}\,d\}$ for $j,\,k=\mathrm{0,}\,\cdots ,\,d-1$. A schematic illustration for the Weyl operator ***W***_31_ on a 4-dimensional Hilbert space is given in Fig. 2. Note that Weyl operators operating on a prime dimensional Hilbert space have *d* distinct eigenvalues (and we can simply state that $s=\mathrm{0,}\,\mathrm{1,}\,\cdots ,\,d-1$) except for ***W***_00_. On the other hand, some Weyl operators of a composite dimension may have repeated eigenvalues. This repetition of eigenvalues restrains us from deriving general forms of our results directly. We circumvent this problem by first presenting our results for the Hilbert space of a prime dimension, and then show that an alternate formulation of our results can be applied to the case of a composite dimensional Hilbert space as well.

A DWC, denoted by ${{\mathscr{N}}}_{{\rm{dw}}}({\boldsymbol{\rho }})$, is a generalization of the Pauli qubit channel^1^, defined in terms of discrete Weyl operators as

5 $${{\mathscr{N}}}_{{\rm{dw}}}({\boldsymbol{\rho }})=\sum _{n\mathrm{=0}}^{d-1}\,\sum _{m\mathrm{=0}}^{d-1}\,{p}_{nm}{{\boldsymbol{W}}}_{nm}{\boldsymbol{\rho }}{{\boldsymbol{W}}}_{nm}^{\dagger }$$

where ***W***_*nm*_ acts on the input state ***ρ*** with probability *p*_*nm*_.

The Holevo capacity of a quantum channel is defined as^6,28^

6 $$\chi ({\mathscr{N}})=\mathop{{\rm{\sup }}}\limits_{\{{p}_{i},{{\boldsymbol{\rho }}}_{i}\}}[S(\sum _{i}\,{p}_{i}{\mathscr{N}}({{\boldsymbol{\rho }}}_{i}))-\sum _{i}\,{p}_{i}S({\mathscr{N}}({{\boldsymbol{\rho }}}_{i}))]$$

where *p*_*i*_ is the *a priori* probability of input state ***ρ***_*i*_; $S({\boldsymbol{\rho }})=-\,{\rm{Tr}}({\boldsymbol{\rho }}\,\mathrm{log}\,{\boldsymbol{\rho }})$ is the von Neumann entropy, and ${\mathscr{N}}({\boldsymbol{\rho }})$ is the output state produced by the action of channel ${\mathscr{N}}$ on the input state ***ρ***. The Holevo capacity corresponds to the maximum rate of classical information when input states are restricted to be separable, i.e., the inputs of the channel are not entangled over multiple uses.

### Lemma 1.

*If the input state of a DWC operating on a d-dimensional Hilbert space is an eigenstate of a d-dimensional Weyl operator* ***W***_*nm*_*, then the output state is diagonal in the eigenbasis of* ***W***_*nm*_.

### *Proof*

. See Methods section.◽

As a consequence of the above Lemma, we can choose the set of input states to be *d* orthogonal eigenvectors of some Weyl operator ***W***_*nm*_, and measure the output in the eigenbasis of ***W***_*nm*_. The uncertainty at the output of the channel in this case is purely classical in nature. In this sense, a DWC is behaving as a classical channel, transitioning a distinguishable state into an unknown but perfectly distinguishable state. We completely characterize the simulated classical channel in terms of channel transition matrix in the following Proposition.

### Proposition 1

*A DWC of a prime dimension d with orthonormal eigenstates of* ***W***_*nm*_ *as the input states behaves as a classical symmetric channel with the following transition matrix*

7 $${{\boldsymbol{T}}}_{nm}=[\begin{array}{cccc}{P}_{1} & {P}_{2} & \cdots  & {P}_{d}\\ {P}_{d} & {P}_{1} & \cdots  & {P}_{d-1}\\ \vdots  & \vdots  & \ddots  & \vdots \\ {P}_{2} & {P}_{3} & \cdots  & {P}_{1}\end{array}],\,\,(n,\,m)\ne (\mathrm{0,}\,0)$$

*where*

8 $${P}_{k}=\sum _{ij:{\omega }^{mi-nj}={\omega }^{k-1}}\,{p}_{ij}\mathrm{.}$$

### *Proof*.

See Methods section.◽

**Figure 3:**
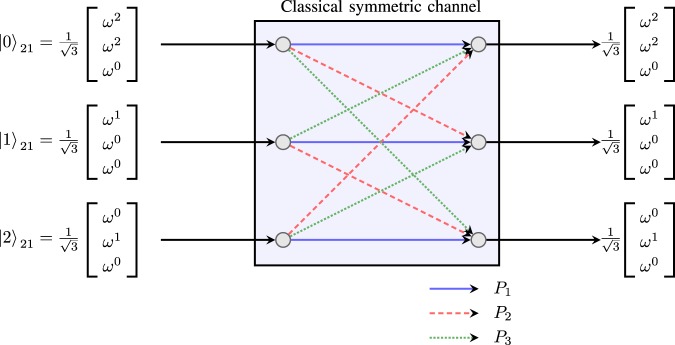
An example DWC for *d* = 3 driven by the eigenstates of ***W***_21_.

As an example, a DWC driven by the eigenstates of ***W***_21_ with *d* = 3 is shown in Fig. 3. In this example, we have ${P}_{1}={p}_{00}+{p}_{21}+{p}_{12}$, ${P}_{2}={p}_{20}+{p}_{11}+{p}_{02}$, and ${P}_{3}={p}_{10}+{p}_{01}+{p}_{22}$.

## Results

Based on the proposition 1, we give the following simple and natural lower bound on the Holevo capacity of a DWC:

### Theorem 1

*The Holevo capacity* $\chi ({{\mathscr{N}}}_{{\rm{dw}}})$ *of the channel in* (5) *with a prime d is bounded as*

9 $$\chi ({{\mathscr{N}}}_{{\rm{dw}}})\ge {\mathrm{log}}_{2}(d)-\mathop{{\rm{\min }}}\limits_{n,m}H({\rm{row}}\,{\rm{of}}\,{{\boldsymbol{T}}}_{nm}),\,\,(n,\,m)\ne \mathrm{(0,}\,\mathrm{0)}$$

where ***T***_*nm*_ *is the channel transition matrix of the (n*, *m) th symmetric channel obtained by fixing the eigenstates of* ***W***_*nm*_ *as the signal states and* $H(\,\cdot \,)$ *is the Shannon entropy*.

### *Proof*

. See Methods section.◽

The restriction on *d* to be a prime number is primarily because the repetition of eigenvalues of ***W***_*nm*_ of a composite *d* does not allow us to construct the channel transition matrix ***T***_*nm*_. The following remark provides us an alternative approach to lower bound the Holevo capacity of DWC of any *d*.

### Remark 1.

*It is straightforward to show that* $H({\rm{row}}\,{\rm{of}}\,{{\boldsymbol{T}}}_{nm})=S({{\mathscr{N}}}_{{\rm{dw}}}(|\lambda \rangle \,{\langle \lambda |}_{nm}))$ *when d is prime*, *where* $|\lambda \rangle {\langle \lambda |}_{nm}$ *is the density matrix of any eigenstate of* ***W***_*nm*_. *Therefore*, *we can equivalently calculate*

10 $$\chi ({{\mathscr{N}}}_{{\rm{dw}}})\ge {\mathrm{log}}_{2}(d)-\mathop{{\rm{\min }}}\limits_{n,m}\,S\,({{\mathscr{N}}}_{{\rm{dw}}}(|\lambda \rangle {\langle \lambda |}_{nm}))$$

*for prime d*. *Then*, *we can extend* (10) *to any d by replacing the optimization on any* ***ρ*** *in* (20) *with the optimization on the eigenstates of* ***W***_*nm*_ *only*.

### Theorem 2.

*Let us define a vector* ${\boldsymbol{\zeta }}({\boldsymbol{p}})\in {{\mathbb{R}}}^{d}$ *such that*

11 $${\boldsymbol{\zeta }}({\boldsymbol{p}})={\boldsymbol{S}}{{\boldsymbol{p}}}^{\downarrow }$$

*where the elements of* ${{\boldsymbol{p}}}^{\downarrow }$ *are the elements of vector* ${\boldsymbol{p}}\in {{\mathbb{R}}}^{{d}^{2}}$ *in descending order; the matrix* ${\boldsymbol{S}}\in {{\mathbb{R}}}^{d\times {d}^{2}}$ *is given by*

12 $$S=[\begin{array}{lllll}{{\bf{1}}}_{d}^{T} & {{\bf{0}}}_{d}^{T} & {{\bf{0}}}_{d}^{T} & \cdots  & {{\bf{0}}}_{d}^{T}\\ {{\bf{0}}}_{d}^{T} & {{\bf{1}}}_{d}^{T} & {{\bf{0}}}_{d}^{T} & \cdots  & {{\bf{0}}}_{d}^{T}\\ {{\bf{0}}}_{d}^{T} & {{\bf{0}}}_{d}^{T} & {{\bf{1}}}_{d}^{T} & \cdots  & {{\bf{0}}}_{d}^{T}\\ \vdots  & \vdots  & \vdots  & \ddots  & \vdots \\ {{\bf{0}}}_{d}^{T} & {{\bf{0}}}_{d}^{T} & {{\bf{0}}}_{d}^{T} & \cdots  & {{\bf{1}}}_{d}^{T}\end{array}]$$

*where* ${(\cdot )}^{T}$ *denotes the transpose operation*, *and* **1**_*d*_ *and* **0**_*d*_ *are all-one and all-zero vectors of d elements*, *respectively*. *Then*, *the Holevo capacity of a DWC is*

13 $$\chi ({{\mathscr{N}}}_{{\rm{dw}}})\le {\mathrm{log}}_{2}(d)-H({\boldsymbol{\zeta }}({\boldsymbol{p}})),$$

*where* ${\boldsymbol{p}}={[{p}_{00}{p}_{01}\cdots {p}_{nm}]}^{T}$, *whose elements are probabilities associated with respective Weyl operators* ***W***_*nm*_.

### *Proof.*

See Methods section.◽

In a *d*-dimensional Hilbert space, *d*^2^ Weyl operators are defined whose indices are given in the form of 2-tuples, e.g., (*i*, *j*). We define a set ${\mathscr{W}}$ that contains all the *d*^2^ indices of defined Weyl operators. We call a set ${\mathscr{D}}$ a *d*-set if all its elements ${{\mathscr{D}}}_{i}$ for $i=\mathrm{0,}\,\cdots ,\,d-1$ are non-overlapping *d* element subsets of ${\mathscr{W}}$

14 $${\mathscr{D}}=\{{{\mathscr{D}}}_{i}|{{\mathscr{D}}}_{i}{\subset }_{d}{\mathscr{W}},{{\mathscr{D}}}_{i}\cap {{\mathscr{D}}}_{j}=\varnothing \,{\rm{for}}\,i\ne j,\,i,\,j=\mathrm{0,}\,\cdots ,\,d-1\}$$

where ${\mathscr{A}}{\subset }_{d} {\mathcal B}$ means that ${\mathscr{A}}$ is a *d*-element subset of ${\mathcal B}$, $\varnothing$ is the empty set, and ${\mathscr{A}}\cap  {\mathcal B}$ gives a set whose elements are the common elements of ${\mathscr{A}}$ and ${\mathcal B}$. In the *d* dimensional Hilbert space, there are

15 $$\frac{1}{d!}\prod _{i=0}^{d-1}(\begin{array}{c}{d}^{2}-id\\ d\end{array})$$

different possible *d*-sets, where

$$(\begin{array}{c}n\\ k\end{array})=\frac{n!}{k!(n-k)!}$$

are the binomial coefficients.

A *d*-set ${\mathscr{D}}$ whose all elements ${{\mathscr{D}}}_{t}$ satisfy the property

16 $$mi-nj\,{\rm{m}}{\rm{o}}{\rm{d}}\,d={k}_{t},\,\,{\rm{\forall }}(i,\,j)\in {{\mathscr{D}}}_{t}$$

for some *n*, *m*, and some constants *k*_*t*_ is called an achievable *d*-set. For example

17 $${\mathscr{D}}=\{\{\mathrm{(0,}\,\mathrm{0),}\,\mathrm{(2,}\,\mathrm{1),}\,\mathrm{(1,}\,\mathrm{2)}\},\,\{\mathrm{(2,}\,\mathrm{0),}\,\mathrm{(1,}\,\mathrm{1),}\,\mathrm{(0,}\,\mathrm{2)}\},\,\{\mathrm{(1,}\,\mathrm{0),}\,\mathrm{(0,}\,\mathrm{1),}\,\mathrm{(2,}\,\mathrm{2)}\}\}$$

is an achievable *d*-set for (*n*, *m*) = (2, 1) but

18 $${\mathscr{D}}=\{\{\mathrm{(0,}\,\mathrm{0),}\,\mathrm{(0,}\,\mathrm{1),}\,\mathrm{(1,}\,\mathrm{2)}\},\{\mathrm{(2,}\,\mathrm{0),}\,\mathrm{(1,}\,\mathrm{1),}\,\mathrm{(2,}\,\mathrm{2)}\},\,\{\mathrm{(1,}\,\mathrm{0),}\,\mathrm{(2,}\,\mathrm{1),}\,\mathrm{(0,}\,\mathrm{2)}\}\}$$

is a *d*-set which is not achievable.

### Theorem 3.

*We arrange the elements of p in nonincreasing order and collect the indices of p*_*nm*_ *while preserving the order to form a d-set*. *The bounds of Theorem 1*, *and Theorem 2 coincide if and only if (resp*. *only if) the obtained d-set is achievable and d is a prime number (resp*. *a composite number)*.

### *Proof.*

See Methods section.◽

### Remark 2.

*If the two bounds coincide*, *we have*

19 $$\chi ({{\mathscr{N}}}_{{\rm{dw}}})={\mathrm{log}}_{2}(d)-\mathop{{\rm{\min }}}\limits_{n,m}H({\rm{row}}\,{\rm{of}}\,{{\boldsymbol{T}}}_{nm}),\,\,(n,\,m)\ne (\mathrm{0,}\,0)\mathrm{.}$$

*However*, *the converse is not true as will be shown by the numerical examples in the next section*.

## Discussion

An efficient approximation for the capacity of classical-quantum channels has previously been discussed without exploiting any special properties of a given channel^24^. For example, it takes 40,154 seconds in order to approximate the Holevo capacity of a Pauli qubit channel with a posteriori error of 1.940 × *10*^−3^. In contrast to existing methods, the average time to calculate the (lower) bound in this paper is of the order 10^−4^ seconds even for large *d* by virtue of the use of special properties of DWCs.

**Figure 4:**
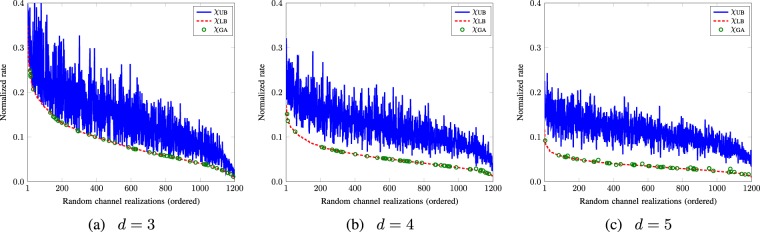
*χ*_UB_, *χ*_LB_, and *χ*_GA_ of random channel realizations (in decreasing order of *χ*_UB_) when *d* = 3, 4, 5.

We have strong numerical evidence that the lower bound is tighter and is saturated more often even when the two bounds do not coincide, as shown in the Fig. 4(a–c) where the upper (*χ*_UB_) and the lower (*χ*_LB_) bounds (normalized by log_2_(*d*)) are plotted for 1200 random channel realizations for *d* = 3, 4, and 5, respectively. In these figures, Holevo capacity by using^23^

20 $$\chi ({{\mathscr{N}}}_{{\rm{dw}}})={\mathrm{log}}_{2}(d)-\mathop{{\rm{\min }}\,}\limits_{{\boldsymbol{\rho }}}S\,({{\mathscr{N}}}_{{\rm{dw}}}({\boldsymbol{\rho }}))$$

with the optimization performed via genetic algorithm (*χ*_GA_) is also presented. Comparison of *χ*_LB_, *χ*_UB_, and *χ*_GA_ shows that the frequency of coincidence of two bounds as well as the frequency of the saturation of the lower bound is higher for the case of *d* = 3.

Our bounds not only ease the requirement of optimization for the calculation of tight bounds for a general DWC, but also allows to recover the analytic expressions for the special cases of DWC. For example, here we recover the analytic expression for the classical capacity of a qudit depolarizing channel using the approach developed above. A quantum depolarizing channel transforms an input state to the output state according to the following map

21 $${{\mathscr{N}}}_{{\rm{d}}}({\boldsymbol{\rho }})=(1-\mu ){\boldsymbol{\rho }}+\mu {\boldsymbol{\pi }}$$

where ${\boldsymbol{\pi }}={{\boldsymbol{I}}}_{d}/d$ is the maximally mixed state on the output Hilbert space. In terms of Weyl operators,

22 $${\boldsymbol{\pi }}=\frac{1}{{d}^{2}}\sum _{n,m\mathrm{=0}}^{d-1}\,{{\boldsymbol{W}}}_{nm}{\boldsymbol{\rho }}{{\boldsymbol{W}}}_{nm}^{\dagger }\mathrm{.}$$

Thus, we can rewrite equation (21) as

23 $${{\mathscr{N}}}_{{\rm{d}}}({\boldsymbol{\rho }})=(1-\mu +\frac{\mu }{{d}^{2}}){\boldsymbol{\rho }}+\frac{\mu }{{d}^{2}}\sum _{n,m\mathrm{=0}(n,m)\ne (\mathrm{0,0})}^{d-1}\,{{\boldsymbol{W}}}_{nm}{\boldsymbol{\rho }}{{\boldsymbol{W}}}_{nm}^{\dagger }\mathrm{.}$$

Therefore

24 $${p}_{00}=1-\mu +\frac{\mu }{{d}^{2}},\,{p}_{nm}=\frac{\mu }{{d}^{2}}\,\forall \,(n,\,m)\ne (\mathrm{0,}\,0)$$

which shows that all *d*-sets (whether achievable or not) are equivalent in terms of summation of *p*_*nm*_ over the elements ${{\mathscr{D}}}_{i}$. Therefore, we can choose an ordering of *p*_*nm*_ such that the condition of Theorem 3 is satisfied and we can use equation (13) to calculate the Holevo capacity. From equation (21) and the output vector of ${\boldsymbol{\zeta }}({\boldsymbol{p}})=({r}_{0},\,{r}_{1},\,\cdots ,\,{r}_{d})$, we see that

25 $${r}_{0}=1-\mu +\frac{\mu }{d},\,{r}_{i}=\frac{\mu }{d}\,{\rm{for}}\,i=\mathrm{1,}\,\cdots ,\,d-1.$$

Thus, the Holevo capacity $\chi ({{\mathscr{N}}}_{{\rm{d}}})$ of this channel is

26 $$\chi ({{\mathscr{N}}}_{{\rm{d}}})={\mathrm{log}}_{2}(d)+(1-\mu +\frac{\mu }{d})\,{\mathrm{log}}_{2}(1-\mu +\frac{\mu }{d})+(d-1)\frac{\mu }{d}\,{\mathrm{log}}_{2}(\frac{\mu }{d})$$

which is equal to the classical capacity of the quantum depolarizing channel^21^.

Additionally, it is easy to see that for a Pauli qubit channel (*d* = 2), there are 3 possible *d*-sets which are all achievable. Therefore, both bounds are exact for the Pauli qubit (and *all its special cases*) channel. With simple algebraic manipulations one can obtain the analytic expressions for the capacities of any of the special cases of the Pauli qubit channel^24^.

From Theorem 3, we can also define special channels for which the two bounds always coincide. This approach gives us a class of quantum channels whose exact Holevo capacity can readily be calculated. We define two such channels here and call them one-parameter depolarizing-like, and two-parameter depolarizing-like channels, respectively.

The one-parameter depolarizing-like channel is defined as

27 $${{\mathscr{N}}}_{{\rm{d1}}}({\boldsymbol{\rho }})=(1-\xi ){{\boldsymbol{W}}}_{ij}{\boldsymbol{\rho }}{{\boldsymbol{W}}}_{ij}^{\dagger }+\xi {\boldsymbol{\pi }},$$

whose exact Holevo capacity is same as (26) with the depolarizing parameter *ξ*.

The two-parameter depolarizing-like channel is

28 $${{\mathscr{N}}}_{{\rm{d2}}}({\boldsymbol{\rho }})=(1-\eta ){{\boldsymbol{W}}}_{ij}{\boldsymbol{\rho }}{{\boldsymbol{W}}}_{ij}^{\dagger }+(1-\kappa ){{\boldsymbol{W}}}_{nm}{\boldsymbol{\rho }}{{\boldsymbol{W}}}_{nm}^{\dagger }+(\eta +\kappa -1){\boldsymbol{\pi }}$$

where $0\le \eta ,\,\kappa \le \mathrm{1,}\,{\rm{and}}\,1\le \eta +\kappa \le 2$. This channel is a further generalization of the one-parameter depolarizing-like channel. The exact Holevo capacity of this channel can readily be calculated by Theorem 3.

In this work we modeled a DWC as a classical symmetric channel for the task of classical communication. Through this modeling, we presented a simple to compute lower bound on the Holevo capacity of a given DWC of an arbitrary dimension. We also gave an intuitive upper bound which coincides with the lower bound under a certain condition. This (sufficient and necessary for a prime *d*, and necessary for a composite *d*) condition, however, is not frequently met despite the frequent convergence of the lower bound to the actual Holevo capacity as shown by the numerical examples. The lower bound was derived by noting the similarity of a quantum channel with a classical channel. An interesting future direction is to find similar cases where the results of classical information theory (which is more mature despite being a special case of quantum information theory) can be applied on the problems of quantum information theory with a little or no modification. Similarly, based on the equality of upper and lower bounds, one can define special channels for which these bounds always coincide. Such characterization of quantum channels can give us a class of channels whose exact Holevo capacity can readily be calculated.

## Methods

### Proof of Lemma 1

Since the DWC is a random unitary channel, the output of the channel is merely the state obtained by randomly applying one of the *d*^2^ Weyl operators on the input. Thus, we need to show that operation of ***W***_*ij*_ on any eigenstate of ***W***_*nm*_ results into an eigenstate of ***W***_*nm*_.

Let

29 $$|\lambda \rangle ={[\begin{array}{c}{\alpha }_{0},{\alpha }_{1},\cdots ,{\alpha }_{d-1}\end{array}]}^{T}$$

be a normalized eigenvector of ***W***_*nm*_ with the corresponding eigenvalue *λ*. From the eigenvalue relation ${{\boldsymbol{W}}}_{nm}|\lambda \rangle =\lambda |\lambda \rangle$, and due to the property 1, we get the following relation among the entries of vector of (29)

30 $${\alpha }_{(m+k){\rm{mod}}d}=\lambda {\omega }^{-nk}{\alpha }_{k},$$

where the eigenvalues *λ* are equidistant points on the unit circle (see Fig. 2). Since we have obtained this relation from the condition of eigenvector, any vector satisfying above relation will be an eigenvector of ***W***_*nm*_.

Now let us consider the effect of any ***W***_*ij*_ on the vector of (29). To this end, we let ${{\boldsymbol{W}}}_{ij}|\lambda \rangle =|\beta \rangle$, and recall property 1 again to write

31 $${{\boldsymbol{W}}}_{ij}|\lambda \rangle =[\begin{array}{c}{\alpha }_{j}\\ {\omega }^{i}{\alpha }_{(j+1){\rm{mod}}d}\\ \vdots \\ {\omega }^{ki}{\alpha }_{(j+k){\rm{mod}}d}\\ \vdots \end{array}]=[\begin{array}{c}{\beta }_{0}\\ {\beta }_{1}\\ \vdots \\ {\beta }_{k}\\ \vdots \end{array}]=|\beta \rangle \mathrm{.}$$

i.e., the *k*th entry of $|\beta \rangle$ is ${\omega }^{ki}{\alpha }_{(j+k){\rm{mod}}d}$.

If the elements of $|\beta \rangle$ exhibit a similar relation as (30), $|\beta \rangle$ is also an eigenvector of ***W***_*nm*_. Repeated use of (30) gives the following relation between the entries of $|\beta \rangle$

32 $${\beta }_{(m+k){\rm{mod}}d}=\lambda {\omega }^{mi-nj}{\omega }^{-nk}{\beta }_{k}$$

which essentially bears the same form as (30); because $\lambda {\omega }^{mi-nj}$ is another eigenvalue of ***W***_*nm*_. Hence the vector $|\beta \rangle ={{\boldsymbol{W}}}_{ij}|\lambda \rangle$ is an eigenvector of ***W***_*nm*_. Since the output state is a statistical mixture of orthonormal eigenstates of ***W***_*nm*_, it is diagonal in the same basis, i.e., in the eigenbasis of ***W***_*nm*_.

### Proof of Proposition 1

Let the input state be an eigestate |*λ*〉 of ***W***_*nm*_ corresponding to the eigenvalue *λ*. From the proof of Lemma 1, the application of ***W***_*ij*_ transforms the input state to the eigenstate of ***W***_*nm*_ corresponding to the eigenvalue $\lambda {\omega }^{mi-nj}$. Since $\omega =\exp (2\pi \iota /d)$, ${\omega }^{mi-nj}$ is always from the set $\{{\omega }^{0},\,{\omega }^{1},\,\cdots ,\,{\omega }^{d-1}\}$. Therefore, we can define,

33 $${P}_{k}=\sum _{ij:{\omega }^{mi-nj}={\omega }^{k-1}}\,{p}_{ij}$$

as the transition probability of |*λ*〉 to the orthogonal state $|\lambda {\omega }^{k-1}\rangle$. We can define the complete set of transition probabilities *P*_*k*_, for $k=\mathrm{1,}\,\mathrm{2,}\,\cdots ,\,d$ only if ***W***_*nm*_ does not have any repeated eigenvalues which is guaranteed only if *d* is prime and $(n,\,m)\ne (\mathrm{0,}\,0)$ (note the similarity between ${\omega }^{mi-nj}$ and the expression for *s* in the definition of eigenvalues).

Furthermore, we notice that the rows of ***T***_*nm*_ are permutations of each other and its columns are permutation of each other. Therefore, ***T***_*nm*_ in (7) defines a classical symmetric channel.

### Proof of Theorem 1

From proposition 1 we know that in this setting DWC acts as a classical symmetric channel. Since the capacity of a symmetric channel with *d* inputs and outputs is given by^2^

34 $${C}_{{\rm{Symmetric}}}={\mathrm{log}}_{{\rm{2}}}(d)-H({\rm{row}}\,{\rm{of}}\,{\rm{transition}}\,{\rm{matrix}}),$$

and we have restricted our input states to be from the eigenstates of Weyl operators, thus

$$\chi ({{\mathscr{N}}}_{{\rm{dw}}})\ge {\mathrm{log}}_{{\rm{2}}}(d)-\mathop{{\rm{\min }}}\limits_{n,m}\,H\,({\rm{row}}\,{\rm{of}}\,{{\boldsymbol{T}}}_{nm}),\,\,(n,\,m)\ne \mathrm{(0,}\,\mathrm{0)}$$

where the condition $(n,\,m)\ne \mathrm{(0,}\,\mathrm{0)}$ along with the condition on *d* to be prime ensures that we can model the given DWC as a classical symmetric channel with the channel transition matrix ***T***_*nm*_ by virtue of Proposition 1.

### Proof of Theorem 2

For a vector ${\boldsymbol{x}}=({x}_{1},\,{x}_{2},\,\cdots ,\,{x}_{n})\in {{\mathbb{R}}}^{n}$, we denote *x*_*i*_ in non-increasing order as

35 $${x}_{[1]}\ge {x}_{[2]}\ge \cdots \ge {x}_{[n]},$$

and denote the vector ${{\boldsymbol{x}}}^{\downarrow }=({x}_{[1]},\,{x}_{[2]},\,\cdots ,\,{x}_{[n]})$ of elements of ***x*** rearranged in nonincreasing order. We denote by ${\boldsymbol{x}}\,\prec \,{\boldsymbol{y}}$ and say ***x*** is majorized by ***y*** if

36 $$\sum _{i\mathrm{=1}}^{k}\,{x}_{[i]}\le \sum _{i\mathrm{=1}}^{k}\,{y}_{[i]}\,\,{\rm{for}}\,k=\mathrm{1,}\,\cdots ,\,n$$

with strict equality when *k* = *n*. For two Hermitian operators ***A*** and ***B***, we denote ${\boldsymbol{A}}\,\prec \,{\boldsymbol{B}}$ if ${\boldsymbol{\lambda }}({\boldsymbol{A}})\,\prec \,{\boldsymbol{\lambda }}({\boldsymbol{B}})$, where ${\boldsymbol{\lambda }}({\boldsymbol{A}})$ is the vector of eigenvalues of ***A***.

Let ***γ*** be the optimal input state, then the Holevo capacity of a DWC is^23^

37 $$\chi ({{\mathscr{N}}}_{{\rm{dw}}})={\mathrm{log}}_{2}(d)-S({{\mathscr{N}}}_{{\rm{dw}}}({\boldsymbol{\gamma }}))\mathrm{.}$$

We can rewrite (13) as

38 $$\chi ({{\mathscr{N}}}_{{\rm{dw}}})\le {\mathrm{log}}_{2}(d)-S({\boldsymbol{\rho }}),$$

where ${\boldsymbol{\rho }}$ is some state with the eigenvalues *q*_*i*_ given by the elements of ${\boldsymbol{\zeta }}({\boldsymbol{p}})$. Comparing (37) and (38), our claim simplifies to

39 $$S({\boldsymbol{\rho }})\le S({{\mathscr{N}}}_{{\rm{dw}}}({\boldsymbol{\gamma }})),$$

or from the Schur concavity of von Neumann entropy^29^

40 $${{\mathscr{N}}}_{{\rm{dw}}}({\boldsymbol{\gamma }})\,\prec \,{\boldsymbol{\rho }},$$

where

41 $${{\mathscr{N}}}_{{\rm{dw}}}({\boldsymbol{\gamma }})=\sum _{n\mathrm{=0}}^{d-1}\,\sum _{m\mathrm{=0}}^{d-1}\,{p}_{nm}{{\boldsymbol{W}}}_{nm}{\boldsymbol{\gamma }}{{\boldsymbol{W}}}_{nm}^{\dagger }\mathrm{.}$$

Eigendecomposition of ${\boldsymbol{\rho }}$ can be written as

42 $${\boldsymbol{\rho }}=\sum _{k\mathrm{=0}}^{d-1}\,{q}_{k}{{\boldsymbol{\rho }}}_{k}$$

43 $$=\sum _{k\mathrm{=0}}^{d-1}\,{q}_{k}{{\boldsymbol{S}}}_{k}{\boldsymbol{\sigma }}{{\boldsymbol{S}}}_{k}^{\dagger }$$

where ${\boldsymbol{\sigma }}$, and ${{\boldsymbol{\rho }}}_{k}$ are some pure states; ${\rm{Tr}}\{{{\boldsymbol{\rho }}}_{i}{{\boldsymbol{\rho }}}_{j}\}=1$ if *i* = *j*, and 0 otherwise; and ***S***_*k*_ are some unitary operators defined by the relation ${{\boldsymbol{S}}}_{k}{\boldsymbol{\sigma }}{{\boldsymbol{S}}}_{k}^{\dagger }={{\boldsymbol{\rho }}}_{k}$. We note that we are free to choose *any* ${\boldsymbol{\rho }}$ as long it has eigenvalues *q*_*k*_. This freedom translates to the choice of ${{\boldsymbol{\rho }}}_{k}$, and hence to ***S***_*k*_.

Equation (40) is true if and only if ^30^, [Theorem 5]

44 $$\sum _{n\mathrm{=0}}^{d-1}\,\sum _{m\mathrm{=0}}^{d-1}\,{p}_{nm}{{\boldsymbol{W}}}_{nm}{\boldsymbol{\gamma }}\,{{\boldsymbol{W}}}_{nm}^{\dagger }=\sum _{i}\,{s}_{i}{{\boldsymbol{U}}}_{i}{\boldsymbol{\rho }}{{\boldsymbol{U}}}_{i}^{\dagger }$$

for some probability vector **s** with elements *s*_*i*_ and some unitary matrices ***U***_*i*_. We write

45 $$\sum _{i}\,{s}_{i}{{\boldsymbol{U}}}_{i}{\boldsymbol{\rho }}{{\boldsymbol{U}}}_{i}^{\dagger }=\sum _{i\mathrm{=0}}^{d-1}\,{s}_{i}{{\boldsymbol{U}}}_{i}(\sum _{k\mathrm{=0}}^{d-1}\,{q}_{k}{{\boldsymbol{S}}}_{k}{\boldsymbol{\sigma }}{{\boldsymbol{S}}}_{k}^{\dagger }){{\boldsymbol{U}}}_{i}^{\dagger }$$

46 $$=\sum _{i\mathrm{=0}}^{d-1}\,\sum _{k\mathrm{=0}}^{d-1}\,{r}_{ik}{{\boldsymbol{U}}}_{i}{{\boldsymbol{S}}}_{k}{\boldsymbol{\sigma }}{{\boldsymbol{S}}}_{k}^{\dagger }{{\boldsymbol{U}}}_{i}^{\dagger }$$

47 $$=\sum _{i\mathrm{=0}}^{d-1}\,\sum _{k\mathrm{=0}}^{d-1}\,{r}_{ik}{{\boldsymbol{U}}}_{i}{{\boldsymbol{S}}}_{k}V{\boldsymbol{\gamma }}{{\boldsymbol{V}}}^{\dagger }{{\boldsymbol{S}}}_{k}^{\dagger }{{\boldsymbol{U}}}_{i}^{\dagger }$$

where we can write ${\boldsymbol{\sigma }}=V{\boldsymbol{\gamma }}{{\boldsymbol{V}}}^{\dagger }$ because both ${\boldsymbol{\sigma }}$ and ***γ*** are pure states, and we can obtain ${r}_{ik}={p}_{ik}$ due to^30^, [Theorem 4]. Without a loss of generality we can assume both ${\boldsymbol{\sigma }}$ and ***γ*** to be the basis states of a basis set each, i.e., ${\boldsymbol{\sigma }}={{\boldsymbol{\sigma }}}_{0}\in { {\mathcal B} }_{{\boldsymbol{\sigma }}}$, and ${\boldsymbol{\gamma }}={{\boldsymbol{\gamma }}}_{0}\in { {\mathcal B} }_{{\boldsymbol{\gamma }}}$. There is also no loss of generality in assuming ${ {\mathcal B} }_{{\boldsymbol{\sigma }}}$ to be the computational basis. Under these assumptions, the unitary ***V*** is the change of basis unitary from ${ {\mathcal B} }_{{\boldsymbol{\gamma }}}$ to the computational basis, i.e.,

48 $${\boldsymbol{V}}=\sum _{j\mathrm{=0}}^{d-1}\,|j\rangle {\langle {\boldsymbol{\gamma }}|}_{j}\mathrm{.}$$

We need to find **U**_*i*_, and **S**_*k*_, such that

49 $${{\boldsymbol{U}}}_{i}{{\boldsymbol{S}}}_{k}{\boldsymbol{V}}={{\boldsymbol{W}}}_{ik}$$

or

50 $${{\boldsymbol{U}}}_{i}{{\boldsymbol{S}}}_{k}={{\boldsymbol{W}}}_{ik}{{\boldsymbol{V}}}^{\dagger }$$

51 $$=\sum _{j\mathrm{=0}}^{d-1}\,{\omega }^{ji}|j\rangle \langle (j+k)\,{\rm{mod}}\,d|\sum _{j^{\prime} \mathrm{=0}}^{d-1}\,|{{\boldsymbol{\gamma }}}_{j^{\prime} }\rangle \langle j^{\prime} |$$

52 $$=\sum _{j\mathrm{=0}}^{d-1}\,\sum _{j^{\prime} \mathrm{=0}}^{d-1}\,{\omega }^{ji}|j\rangle \langle j^{\prime} |\,\langle (j+k)\,{\rm{mod}}\,d||{{\boldsymbol{\gamma }}}_{j^{\prime} }\rangle \mathrm{.}$$

Choosing

53 $${{\boldsymbol{S}}}_{k}=\sum _{j^{\prime} \mathrm{=0}}^{d-1}|{{\boldsymbol{\gamma }}}_{(j^{\prime} -k){\rm{mod}}d}\rangle \langle j^{\prime} |,\,{\rm{and}}\,{{\boldsymbol{U}}}_{i}=\sum _{j\mathrm{=0}}^{d-1}\,{\omega }^{ji}|j\rangle \langle j|$$

satisfies the above product (the indexing of *j* and of ${{\boldsymbol{\gamma }}}_{j}$ is arbitrary except for *j* = 0), as well as the orthogonality of ${{\boldsymbol{\rho }}}_{k}={{\boldsymbol{S}}}_{k}{\boldsymbol{\sigma }}{{\boldsymbol{S}}}_{k}^{\dagger }$. Therefore, (13) is an upper bound on the Holevo capacity of a DWC.

### Proof of Theorem 3

We first observe that the condition on the summation in (8) for the lower bound, and the condition on a *d*-set to be achievable (16) are essentially the same and result in the same *d*-element partitioning and ordering of *p*_*nm*_. Thus, in a prime dimension *d*, every achievable *d*-set corresponds to a classical symmetric channel that can be simulated by DWC for some *n*, *m*.

On the other hand, the upper bound is obtained by ordering the elements of *p*_*nm*_ in a nonincreasing order. Therefore, the achievability of the *d*-set formed by the indices of *p*_*nm*_ when the *p*_*nm*_ are arranged in a nonincreasing order is sufficient for the existence of a simulated classical symmetric channel of prime dimension that achieves the upper bound. Similarly, since the correspondence of achievable *d*-sets to a simulated classical symmetric channel is bijective, therefore the conincidence of two bounds necessarily implies the achievability of the *d*-set formed above.

For a composite *d*, the correspondence between the simulated classical symmetric channel to the achievable *d*-sets is injective-only. Therefore the above condition is necessary but no longer sufficient for the coincidence of two bounds.

## Electronic supplementary material

Supplementary Material
