# Supplementary material for: Holevo Capacity of Discrete Weyl Channels
Source: Sci Rep. 2018 Nov 29;8:17457. doi: 10.1038/s41598-018-35777-7 (PMC6265333; doi:10.1038/s41598-018-35777-7)
Supplement: Supplementary file 1 — Supplementary Material [file 41598_2018_35777_MOESM1_ESM.pdf]

Supplementary Material: Holevo Capacity of Discrete Weyl Channels

Junaid ur Rehman[1], Youngmin Jeong[1,*], Jeong San Kim[2], and Hyundong Shin[1,*]

[1]Department of Electronic Engineering, Kyung Hee University, 1732 Deogyeong-daero, Giheung-gu, Yongin-si, Gyeonggi-do, 17104 Korea.
[2]Department of Applied Mathematics and Institute of Natural Sciences, Kyung Hee University, 1732 Deogyeong-daero, Giheung-gu, Yongin-si, Gyeonggi-do, 17104 Korea.
[*]Correspondence and requests for materials should be addressed to Y.J and H.S (email: yjeong@khu.ac.kr; hshin@khu.ac.kr)

This supplementary material provides detailed derivations of the eigenvalues of discrete Weyl operators. We reiterate a part of the main text to make the supplementary material more explicit and self-contained.

Eigenvalues of Discrete Weyl Operators

Discrete Weyl operators are a non-Hermitian generalization of Pauli operators for dimension d[1]. A Weyl operator $\boldsymbol{W}_{nm}$ on the d-dimensional Hilbert space is defined as[2]

$$\boldsymbol{W}_{nm} = \sum_{k=0}^{d-1} \omega^{kn} \left|k\right\rangle \left\langle (k+m) \mod d\right| \tag{S1}$$

for $n,m = 0,1,\cdots,d-1$; $\omega = \exp(2\pi\imath/d)$; and $|k\rangle$ is the kth basis vector in the computational basis (for notational convenience, the indexing of entries of vectors and matrices start from 0).

The eigenvalues of a Weyl operator $\boldsymbol{W}_{nm}$ are given by

$$\lambda_s = \omega^{mn\frac{(d-1)}{2}+s} \tag{S2}$$

where $s \in \{(mk-nj) \mod d\}$ for $j,k = 0,\cdots,d-1$. Note that Weyl operators operating on a prime dimensional Hilbert space have d distinct eigenvalues (and we can simply state that $s = 0,1,\cdots,d-1$) except for $\boldsymbol{W}_{00}$. On the other hand, some Weyl operators of a composite dimension may have repeated eigenvalues.

Sketch of the Proof

The sketch of the proof is as follows. We use a previously known result[3] to obtain an equation that has exactly ℓ distinct solutions, and all these ℓ solutions are the eigenvalues of a Weyl operator $\boldsymbol{W}_{nm}$. Then, we show that (S2) generates all ℓ solutions of the said equation.

It is shown in [3, Theorem 4] that the distinct eigenvalues $\tilde{\nu}_k$ (upto an appropriate phase factor) of Weyl operators operating on a d-dimensional Hilbert space are given by

$$\tilde{\nu}_k = \exp(2\pi\imath k/\ell), \tag{S3}$$

for $0 \le k \le \ell-1$, where ℓ is either equal to d or is some divisor of d. Let p' be the said phase factor, then the distinct eigenvalues ν_k (with the exact phase) of a Weyl operator $\boldsymbol{W}_{nm}$ are given by

$$\nu_k = p' \exp(2\pi\imath k/\ell). \tag{S4}$$

Note that (S4) are ℓth roots of some $p = (p')^{\ell}$. In the following we derive some properties of ℓ, and obtain an explicit expression for p. Then, we show that (S2) generates the same eigenvalues as (S4) with the correct phase p'.

Determining ℓ and p

If an operator $\boldsymbol{A}$ has eigenvalues $\{\mu_1,\mu_2,\cdots,\mu_n\}$, then the eigenvalues of $\boldsymbol{A}^x$ are $\{\mu_1^x,\mu_2^x,\cdots,\mu_n^x\}$[4]. Since the eigenvalues of $\boldsymbol{W}_{nm}$ are all ℓth roots of p, $(\boldsymbol{W}_{nm})^{\ell}$ has only one eigenvalue i.e., p. Combining this fact with the fact that $\boldsymbol{W}_{nm}$ are full rank matrices, and any similarity transform of identity results into identity, we deduce that

$$(\boldsymbol{W}_{nm})^{\ell} = pI. \tag{S5}$$

Furthermore, since there does not exist any $\ell' < \ell$, such that $(\nu_k)^{\ell'} = p''$, for some p'' and for all $0 \le k \le \ell - 1$, therefore ℓ is the smallest number such that $(\boldsymbol{W}_{nm})^{\ell}$ is proportional to I. The explicit expression for $(\boldsymbol{W}_{nm})^q$ for any integer q is obtained in the following Lemma.

Lemma 1. *For any integer q and a Weyl operator $\boldsymbol{W}_{nm}$*

$$(\boldsymbol{W}_{nm})^q = \sum_{k=0}^{d-1} \omega^{\left(qk+\frac{q(q-1)}{2}m\right)n} |k\rangle \langle (k+qm) \bmod d| . \tag{S6}$$

Proof. We prove this result by induction. Since Weyl operator $\boldsymbol{W}_{nm}$ operating on a d-dimensional Hilbert space is defined as

$$\boldsymbol{W}_{nm} = \sum_{k=0}^{d-1} \omega^{kn} |k\rangle \langle (k+m) \bmod d| , \tag{S7}$$

we have

$$(\boldsymbol{W}_{nm})^2 = \sum_{k=0}^{d-1} \omega^{kn} |k\rangle \langle (k+m) \bmod d| \sum_{j=0}^{d-1} \omega^{jn} |j\rangle \langle (j+m) \bmod d| , \tag{S8}$$

$$= \sum_{k=0}^{d-1} \omega^{(2k+m)n} |k\rangle \langle (k+2m) \bmod d| , \tag{S9}$$

where we have used the orthonormality of basis vectors.

Now we assume that (S6) is true for $(\boldsymbol{W}_{nm})^{q-1}$, i.e.,

$$(\boldsymbol{W}_{nm})^{q-1} = \sum_{k=0}^{d-1} \omega^{\left((q-1)k+\frac{(q-1)(q-2)}{2}m\right)n} |k\rangle \langle (k+(q-1)m) \bmod d| , \tag{S10}$$

then,

$$(\boldsymbol{W}_{nm})^{q-1}\boldsymbol{W}_{nm} = \sum_{k=0}^{d-1} \omega^{\left((q-1)k+\frac{(q-1)(q-2)}{2}m\right)n} |k\rangle \langle (k+(q-1)m) \bmod d| \sum_{j=0}^{d-1} \omega^{jn} |j\rangle \langle (j+m) \bmod d| , \tag{S11}$$

$$= \sum_{k=0}^{d-1} \omega^{\left((q-1)k+\frac{(q-1)(q-2)}{2}m+k+(q-1)m\right)n} |k\rangle \langle (k+(q-1)m+m) \bmod d| , \tag{S12}$$

$$= \sum_{k=0}^{d-1} \omega^{\left(qk+\frac{q(q-1)}{2}m\right)n} |k\rangle \langle (k+qm) \bmod d| , \tag{S13}$$

which recovers the expressions (S7), (S9), and (S10) for $q = 1, 2$, and $q-1$, respectively, and proves the statement of our lemma. □

In the following Lemma we show that ℓ is always a divisor of d.

Lemma 2. *For a d-dimensional Weyl operator $\boldsymbol{W}_{nm}$, the minimum $\ell \in \{1, 2, \cdots, d\}$ such that $(\boldsymbol{W}_{nm})^{\ell} = pI$ is always a divisor of d.*

Proof. Assume that ℓ is not a divisor of d, then

$$d = e\ell + r, \tag{S14}$$

for some e and $0 < r < \ell$. Then,

$$(\boldsymbol{W}_{nm})^d = (\boldsymbol{W}_{nm})^{(e\ell+r)} , \tag{S15}$$

$$= (\boldsymbol{W}_{nm})^{e\ell} (\boldsymbol{W}_{nm})^r , \tag{S16}$$

$$= p^e I (\boldsymbol{W}_{nm})^r , \tag{S17}$$

which is not proportional to identity since ℓ is the least power such that $(\boldsymbol{W}_{nm})^{\ell}$ is proportional to identity, and $0 < r < \ell$. It is easy to see from Lemma 1 that $(\boldsymbol{W}_{nm})^d$ is always proportional to identity. Therefore, our starting assumption that ℓ is not a divisor of d results into a contradiction and is wrong. Hence, the minimum ℓ such that $(\boldsymbol{W}_{nm})^{\ell} = pI$ is always a divisor of d. □

Now writing the condition (S5) explicitly in the Dirac's notation, i.e.,

$$\sum_{k=0}^{d-1} \omega^{\left(\ell k+\frac{\ell(\ell-1)}{2}m\right)n} |k\rangle \langle (k+\ell m) \bmod d| = \sum_{k=0}^{d-1} p\,|k\rangle \langle k|, \tag{S18}$$

we obtain

$$p = \omega^{\left(\ell k+\frac{\ell(\ell-1)}{2}m\right)n}, \tag{S19}$$

and the following conditions on ℓ

$$\ell m = ad \quad \because (k+\ell m) \bmod d = k, \tag{S20}$$

$$\ell n = bd \quad \because \omega^{\left(\ell k+\frac{\ell(\ell-1)}{2}m\right)n} = p \;\forall k, \tag{S21}$$

where $0 \leq a \leq m$ and $0 \leq b \leq n$ are some positive integers. Note that since ℓ is the smallest number such that $(\boldsymbol{W}_{nm})^{\ell}$ is proportional to identity, therefore ℓ is the smallest number satisfying conditions (S20) and (S21). It is easy to see from the minimality of ℓ that a, b, and ℓ are relatively prime (they do not have a common divisor greater than 1).

Using (S20) and (S21), we can simplify (S19) as

$$p = \omega^{\frac{\ell(\ell-1)}{2}mn} \tag{S22}$$

$$= \begin{cases} \omega^{-\ell nm/2}, & \text{when } \ell \text{ is even} \\ \omega^{u\ell nm} = 1, & \text{when } \ell \text{ is odd,} \end{cases} \tag{S23}$$

where $u = (\ell-1)/2$ is an integer.

Therefore, the eigenvalues of $\boldsymbol{W}_{nm}$ are the ℓth roots of p, i.e., they are ℓ distinct numbers that satisfy

$$p - x^{\ell} = 0, \tag{S24}$$

where p is given by (S23) and ℓ satisfies the conditions (S20) and (S21). In the next section we show that (S2) generates ℓ distinct numbers that satisfy (S24).

Generating ℓth Roots of p by (S2)

As stated in the main text (given by (S2) here) that the eigenvalues of any $\boldsymbol{W}_{nm}$ on a d-dimensional Hilbert space are given by

$$\lambda = \omega^{nm\frac{(d-1)}{2}+(mk-nj)}, \tag{S25}$$

for $j, k = 0, \cdots, d-1$; where we have substituted the variable s with its expression for the clarity in the upcoming calculations. The validity of this expression can be established by showing that it generates all ℓth roots of p. We show this in two steps, i) every value generated by (S25) satisfies (S24), i.e., (S25) generates ℓth roots of p, and ii) it generates exactly ℓ unique values, i.e., it generates *all* ℓth roots of p.

Note that

$$\lambda^{\ell} = \omega^{\ell nm\frac{(d-1)}{2}+\ell(mk-nj)}, \tag{S26}$$

$$= \omega^{\ell nm\frac{(d-1)}{2}}, \tag{S27}$$

where the last equality follows from conditions (S20) and (S21) and holds for every integer k and j.

Continuing with the last expression while considering the case when ℓ is even, we have

$$\omega^{\ell nm\frac{(d-1)}{2}} = \omega^{\frac{\ell nmd}{2}} \omega^{-\frac{\ell nm}{2}}, \tag{S28}$$

$$= \omega^{-\frac{\ell nm}{2}}, \tag{S29}$$

$$= p, \tag{S30}$$

where $\omega^{\frac{\ell nmd}{2}} = 1$, because $\ell/2$ is an integer and the exponent term is an integer multiple of d.

The case when ℓ is odd can be further divided into the following two cases

i) When d is odd, $(d-1)/2 = v$ is an integer, and using (S21), we write

$$\omega^{\ell nm\frac{(d-1)}{2}} = \omega^{bdmv}, \tag{S31}$$
$$= 1. \tag{S32}$$

ii) When d is even, the right hand sides of both (S20) and (S21) are even. Since ℓ is odd, therefore both m and n must be even for the equalities to hold. Hence, $m/2 = w$ is an integer, and we write

$$\omega^{\ell nm\frac{(d-1)}{2}} = \omega^{bdw(d-1)}, \tag{S33}$$
$$= 1. \tag{S34}$$

Hence, we have shown that $\lambda^{\ell} = p$ for every integer j and k, i.e., every value generated by (S25) is indeed an eigenvalue of $\boldsymbol{W}_{nm}$. What is left to prove now is that it generates all the distinct eigenvalues of $\boldsymbol{W}_{nm}$. This can be proven simply by showing that (S25) generates exactly ℓ distinct values.

Let N be the total number of distinct values generated by (S25). Then, it is trivially true that $N \leq \ell$ since there are no more than ℓ distinct numbers that satisfy (S24). We need to show that (S25) generates at least ℓ distinct values. Since $nm(d-1)/2$ in the exponent of (S25) is a constant for any given n, m, and d, we only need to show that $(mk - nj) \bmod d$, for $0 \leq j, k \leq d-1$ generates at least ℓ distinct values. Let

$$(mj - nk) \bmod d = hc, \tag{S35}$$

where $c = d/\ell$, then showing the existence of $0 \leq j, k \leq d-1$ for every $0 \leq h \leq \ell - 1$ shows that (S25) generates at least ℓ distinct values (because all hc are distinct in the defined range). We substitute the values of m and n from (S20) and (S21) to obtain the equivalent condition

$$\frac{ad}{\ell}j - \frac{bd}{\ell}k = h\frac{d}{\ell} \tag{S36}$$
$$(aj - bk) \bmod \ell = h. \tag{S37}$$

Since a, b, and ℓ are relatively prime, the existence of $0 \leq j, k \leq d-1$, in (S37) for every $0 \leq h \leq \ell - 1$ is guaranteed by the Bézout's identity[5]. Therefore, (S25) generates at least ℓ distinct numbers. Since there are no more than ℓ distinct numbers that satisfy (S24), we deduce that (S25) generates exactly ℓ distinct values.

In this supplementary material, we have shown that (S2) is a generator of eigenvalues of $\boldsymbol{W}_{nm}$, for $n, m = 0, 1, \cdots, d-1$, and for any $d \geq 2$. This expression is particularly useful since it only depends on n, m, and d, and does not require any tedious calculations. Furthermore, it was previously known[3] that the eigenvalues of Weyl operators are given by the ℓth roots of some complex number $p, |p| = 1$. However, specific properties of ℓ and p were not known. On our way to proving the validity of (S2), we have derived certain properties of ℓ, and an explicit expression for p. These new insights can be helpful in simply deriving some properties of Weyl operators. For example, it is straightforward to show that any Weyl operator (except for $\boldsymbol{W}_{00}$) operating on a prime dimensional Hilbert space does not have any repeated eigenvalue (i.e., $\ell = d$) by using conditions (S20) and (S21) etc.

References

1. Bertlmann, R. A. & Krammer, P. Bloch vectors for qudits. *J. Phys. A* **41**, 235303 (2008).

2. Weyl, H. Quantenmechanik und gruppentheorie. *Zeitschrift für Physik* **46**, 1–46 (1927).

3. Baumgartner, B., Hiesmayr, B. & Narnhofer, H. A special simplex in the state space for entangled qudits. *J. Phys. A* **40**, 7919 (2007).

4. Strang, G. *Introduction to Linear Algebra* (Wellesley-Cambridge Press Wellesley, MA, 2016), 5 edn.

5. Tignol, J.-P. *Galois' Theory of Algebraic Equations* (World Scientific, Singapore, 2001).
